# Application of acoustic radiation force impulse elastography combined with serum markers in Child-Pugh grading

**DOI:** 10.6061/clinics/2020/e1670

**Published:** 2020-09-03

**Authors:** Linglin Wei, Zhen Ye, Zhongtao Bao, Xiang Xu, Xiaoyu Lin, Ling Chen

**Affiliations:** I Department of Ultrasound, The First Affiliated Hospital of Fujian Medical University, Fuzhou, China.; II Liver Center, The First Affiliated Hospital of Fujian Medical University, Fuzhou, China.

**Keywords:** Cirrhosis, Chronic Hepatitis B, Child-Pugh, Acoustic Radiation Force Impulse, Serum Marker

## Abstract

**OBJECTIVES:**

Acoustic radiation force impulse (ARFI) elastography, the aspartate aminotransferase-to-alanine aminotransferase ratio (AAR), aspartate aminotransferase-to-platelet ratio index (APRI), and the fibrosis-4 (FIB-4) index are widely used to assess liver fibrosis. However, efficacies of these methods in the evaluation of hepatic functional reserve remain unclear. In this study, we investigated the relationship between ARFI elastography combined with either AAR, APRI, or FIB-4 index and Child-Pugh (CP) class for the evaluation of hepatic functional reserve in patients with chronic hepatitis B (CHB)-related cirrhosis.

**METHODS:**

The shear wave velocities of 104 patients with clinically confirmed CHB-related cirrhosis were determined using the ARFI; and clinical serum markers (e.g. ALT, AST, PLT) were used to calculate the AAR, APRI, and FIB-4 index. Cirrhosis patients were scored according to their CP class. The ARFI, AAR, APRI, and FIB-4 index were compared with the CP class. The efficacy of each indicator in diagnosis was analyzed using the receiver operating characteristic (ROC) curve and the ARFI combined with either the AAR, APRI, or FIB-4 index, which is used to predict decompensated cirrhosis.

**RESULTS:**

No significant differences were observed in gender and age among CP classes A, B, and C patients (*p*>0.05). The ARFI values and the AAR, APRI, and FIB-4 index of patients with CP classes A, B, and C were significantly different (*p*<0.05). With an increasing CP class, the ARFI, AAR, APRI, and FIB-4 values increased. The correlation between the ARFI and the CP class was stronger than that between the AAR, APRI, and FIB-4 index and the CP class. The area under the ROC curve for the diagnosis of decompensated cirrhosis using the ARFI was 0.841, which was higher than that for the AAR, APRI, and FIB-4 index. According to the area under the curve results, no significant differences were found when the ARFI was combined with either the AAR, APRI, or FIB-4 index and when the ARFI alone was used.

**CONCLUSIONS:**

The ARFI value has a strong correlation with the CP class. Therefore, ARFI elastography complements CP class in the assessment of the hepatic functional reserve in patients with CHB-related cirrhosis.

## INTRODUCTION

Hepatitis B virus (HBV) infection is the most common cause of liver disease in China. About 97 million people are HBV carriers, among which more than 20 million suffer from active chronic HBV infection (1,2). Persistent HBV infection may cause chronic hepatitis B (CHB), cirrhosis, and/or hepatocellular carcinoma. Patients may exhibit clinical symptoms including ascites, esophageal varices, splenomegaly, hepatic encephalopathy (HE), and liver failure. Hepatic function plays an important role in predicting the development and prognosis of patients with cirrhosis. Therefore, accurately determining the hepatic functional reserve using auxiliary methods is very important.

The most commonly used method for the assessment of the hepatic functional reserve in the clinic is the Child-Pugh (CP) class. It consists of five clinical features including ascites, HE, total bilirubin, albumin, and prothrombin time or international normalized ratio (INR) (3), and is used to predict the prognosis of patients with CHB or cirrhosis (4,5). However, there are several disadvantages of using the CP scores for this purpose. The presence of ascites and HE indicators is subjective. The use of diuretics and lactulose may lead to uncertainty. The INR does not sufficiently reflect coagulopathy and liver function in cirrhosis (6) and interlaboratory variation may exist in INR values (7). Thus, it is necessary to explore other parameters for the evaluation of the hepatic functional reserve of patients with CHB.

Recently, a number of alternative methods including the use of serum markers and acoustic radiation force impulse (ARFI) elastography based on ultrasound (8-10) were studied for the assessment of liver fibrosis. ARFI imaging is a non-invasive ultrasound elastography technique used to detect the degree of liver fibrosis by measuring liver stiffness, especially for the diagnosis of cirrhosis (11-13). By short-duration acoustic radiation forces, the region of interest localizes the displacements without external compression, and thus the operator dependency is reduced (14). Indirect biomarkers such as the aspartate aminotransferase-to-platelet ratio index (APRI), aspartate aminotransferase-to-alanine aminotransferase ratio (AAR), and fibrosis-4 (FIB-4) index have been investigated for the evaluation of the degree of liver fibrosis (15). Studies have shown that the APRI and FIB-4 are good indicators of the degree of liver fibrosis during CHB (16,17), and the latest World Health Organization guidelines for hepatitis B indicate that in countries or regions with limited resources, the APRI is recommended for the evaluation of liver fibrosis (18). The degree of fibrosis is closely related to the hepatic functional reserve and as the degree increases, the number of functional hepatocytes decreases. Subsequently, the degree of cirrhosis is also aggravated, and the hepatic functional reserve deteriorates.

However, the efficacy of the ARFI combined with either the APRI, AAR, or FIB-4 index in the evaluation of hepatic functional reserve during CHB-related cirrhosis remains unclear. Previous studies reported that liver stiffness is correlated with the CP class (19,20). Thus, we compared and analyzed the correlation between the ARFI, AAR, APRI, and FIB-4 and the CP class in CHB-related cirrhosis patients. We also discussed the clinical application value of the ARFI combined with serum markers in the evaluation of the hepatic functional reserve.

## MATERIALS AND METHODS

### Patients

The study was approved by the local ethics committee, and signed informed consent was provided before participation. A total of 107 patients with CHB-related cirrhosis were enrolled in this study from August 2017 to July 2018. Cirrhosis was diagnosed based on the results of histological examination of liver tissue or based on clinical, laboratory, and radiological examination results. Patients with cirrhosis caused by hepatitis C, alcohol, drugs, or other factors; those with suspected or confirmed primary liver cancer or malignant tumors at other sites; those who had undergone spleen resection and other surgery to relieve portal hypertension symptoms; those with a body mass index ≥30 kg/m^2^; and patients who did not cooperate during the procedures were excluded from the study. The ARFI and serum markers (liver function, blood routine, and coagulation function) were evaluated in all 107 patients before treatment.

### Examination

ARFI imaging was performed using the Siemens Acuson S2000TM ultrasound system with a 4C1 convex array probe. All patients underwent ARFI examination performed by a senior physician blinded to the clinical information of patients throughout the study. During the examination, patients were placed in the slight left lateral position and the right upper limb was raised. The sampling frame was placed in the right lobe of the liver, 1-3 cm below the liver capsule, avoiding the visible structure of the large blood vessels and bile ducts. The UPDATE button was clicked to obtain the shear wave velocity (m/s) in the breath-holding state, and was repeated 10 times to calculate the median value. When a value was significantly different from the others due to the patient’s respiratory motion or adjacent small blood vessels in the sample frame, the number of measurements was increased by more than 15 times. Successful reliable measurements were characterized by: median value of liver stiffness measurements with a success rate ≥60% and an interquartile range/median ratio (IQR/M) <30% (21), and these measurements were included in the statistical analyses.

### CP class and serum markers

Information on HE, ascites, bilirubin, albumin, and prothrombin time were collected, and the CP class was evaluated using the CP scoring system by a senior doctor who was engaged in research on liver disease and was blinded to the ARFI examination results throughout the study. According to their CP scores, patients were classified as follows: CP class A, 5-6; B, 7-9; and C, 10-15. The APRI, AAR, and FIB-4 were calculated as follows:

APRI=[(AST/ULN) 100]/PLT (10^9^/L),

where ULN is the upper limit of normal (40U/L for men and 35U/L for women in our laboratory).

AAR=AST(U/L)/ALT(U/L), FIB-4=age (years)×AST(U/L)/[PLT(10^9^/L)×ALT(U/L)]^1/2^

### Statistical analysis

All data were analyzed using SPSS 19.0 and MedCalc software, and numerical variables are presented as the mean±standard of deviation or M (IQR). For variables with normal distribution, differences in the ARFI, AAR, APRI, and FIB-4 index among the CP class groups were analyzed using one-way analysis of variance; otherwise the Kruskal-Wallis test was used. The correlation of the ARFI, AAR, APRI, and FIB-4 index with the CP class was analyzed using Spearman’s correlation. MedCalc software was used to draw the receiver operating characteristic (ROC) curve to evaluate the performance of the ARFI, AAR, APRI, and FIB-4 index in the diagnosis of decompensated cirrhosis and to determine the optimal cut-off value (maximum of the sum of sensitivity and specificity). The combined prediction model (ARFI+AAR, ARFI+APRI, and ARFI+FIB-4 index) was constructed using logistic regression analysis. The ROC curve was drawn using the predicted probability and the accuracy of decompensated cirrhosis diagnosis using each combined model was assessed using the area under the curve (AUC). The AUC in each combined model was compared using the DeLong test. *p*<0.05 was considered to be statistically significant.

## RESULTS

### Gender and age of patients in each CP class group

A total of 107 patients were enrolled; however, three were excluded because their IQR/M ARFI values were >30%. Thus, the analysis involved 104 patients, which comprised of 80.9% (n=84) men and 19.2% (n=20) women. The ages of patients ranged from 25 to 80 years, with an average age of 52.73±11.93 years. There were 24, 45, and 35 cases of CP classes A, B, and C, respectively. There were no significant differences in gender and age (*p*>0.05; Table 1).

### Analysis of the correlation between the ARFI, AAR, APRI, and FIB-4 index and the CP class

The ARFI, AAR, APRI, and FIB-4 index were significantly different for patients with different CP classes. The ARFI, AAR, APRI, and FIB-4 values increased with increasing CP class. There were significant differences in the four indicators between CP class A patients and CP classes B and C patients; however, no statistical difference was found between CP classes B and C patients. The correlation between the ARFI value (r_s_=0.457, *p*=0.000) and the CP class was stronger than that between the AAR (r_s_=0.301, *p*=0.002), APRI (r_s_=0.23, *p*=0.019), and FIB-4 index (r_s_=0.354, *p*=0.000; Table 2) and the CP class.

### Predictive value of the ARFI, AAR, APRI, and FIB-4 index for the non-invasive diagnosis of decompensated cirrhosis

**Figure 1:**
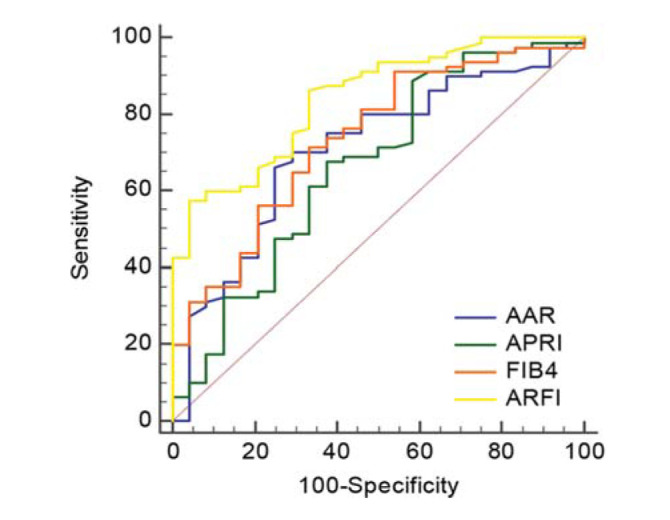
Receiver operating characteristic (ROC) curves of the ARFI, AAR, APRI, and FIB-4 index for the diagnosis of decompensated cirrhosis.

The value of the ARFI, AAR, APRI, and FIB-4 index in predicting the hepatic functional reserve was evaluated using the ROC curve. The optimal cut-off value, sensitivity and specificity, positive predictive value (PPV), and negative predictive value (NPV) of the ARFI, AAR, APRI, and FIB-4 index for the diagnosis of patients with decompensated cirrhosis (at least CP class B) are shown in Figure 1 and Table 3. The AUC of the ARFI for the diagnosis of decompensated cirrhosis was 0.841, which was significantly higher than that of the AAR (0.707), APRI (0.664), and FIB-4 index (0.736). The cut-off values for the ARFI, AAR, APRI, and FIB-4 were 2.99, 1.25, 0.84, and 5.00, respectively.

### Value of ARFI combined with the AAR, APRI, or FIB-4 index in the diagnosis of decompensated cirrhosis

**Figure 2:**
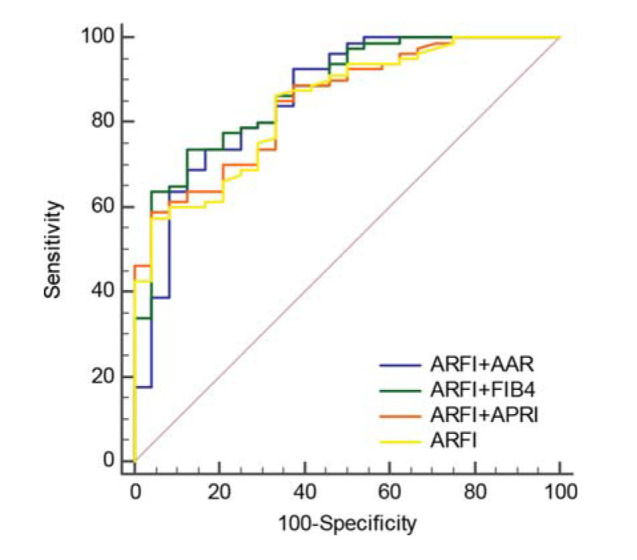
Receiver operating characteristic (ROC) curves of ARFI combined with the AAR, APRI, or FIB-4 index for the diagnosis of decompensated cirrhosis.

A logistic regression model was established, and the predictive value of combined indicators in the diagnosis of decompensated cirrhosis was analyzed using the ROC curve. The sensitivity and specificity, PPV, and NPV are shown in Figure 2 and Table 4. The AUC of the combined indicators for the diagnosis of decompensated cirrhosis were ARFI+AAR, 0.858; ARFI+APRI, 0.847; and ARFI+FIB-4, 0.874. According to the AUC results, no significant difference was found between ARFI+AAR (Z=0.608, *p*=0.543), ARFI+APRI (Z=0.851, *p*=0.395), and ARFI+FIB-4 (Z=1.469, *p*=0.142) and ARFI alone (Table 5).

## DISCUSSION

The results showed that the ARFI, AAR, APRI, and FIB-4 index all increased when the CP class increased. As the patient’s condition deteriorated, the degree of liver fibrosis and cirrhosis also increased, which is expected to provide a reference value for predicting the progress and prognosis of patients with cirrhosis. In addition, the correlation between the ARFI value and the CP class was stronger than that between the AAR, APRI, and FIB-4 index and the CP class, indicating that the predictive value of ARFI was significantly better than that of the other markers. These results were partly consistent with those of previous studies that showed that the higher the CP class, the more severe the liver function damage and the worse the liver functional reserve. Hashimoto et al. (22) found that as the liver fibrosis and cirrhosis worsened, the CP class increased. Kim et al. (23) compared the average ARFI value with the CP score, and found that they were significantly correlated (Spearman’s correlation coefficient, r_s_=0.459).

The comparison of ARFI values for the corresponding CP class groups showed that the ARFI values of CP class A patients were significantly lower than those of CP classes B and C patients, similar to the results of Bota et al. (24). The AAR, APRI, and FIB-4 index of CP class A patients were significantly lower than those of CP classes B and C patients. The reason for the limited ability to distinguish between classes B and C patients may be because the scores of CP class C patients (about 91%) were mainly between 10 and 11 and these scores were similar to those of CP class B patients, which resulted in an overlap in the liver fibrosis degree.

Compared to the general population, the mortality rate of patients with compensated cirrhosis is five-fold higher, whereas the mortality rate of patients with decompensation is 10-fold higher (25). Studies by Kamath et al. (26) showed that the mortality rate of cirrhosis increased with increasing CP grade. The mortality rate of CP grade C was 51%, and the accuracy of predicting short-term mortality was 84%. Some scholars have systematically reviewed the literature on the prognosis of patients with compensated cirrhosis and found that the median survival time was 12 years; however, that of patients with decompensated cirrhosis was two years. The CP class is an independent influencing factor for predicting the prognosis of cirrhosis patients (27). The performance of the four indicators, the ARFI, AAR, APRI, and FIB-4 index, in the diagnosis of decompensated CHB-related cirrhosis was evaluated and all four had specific diagnostic values. Among the ROC curves, the APRI and ARFI ROC curves had the minimum AUC (0.664) and maximum AUC (0.841), respectively, which showed that the ARFI was the best indicator for predicting and identifying compensatory and decompensated cirrhosis among the four indicators. However, the ARFI was more suitable for predicting decompensated cirrhosis rather than its exclusion. For example, using the cut-off value of 2.99 m/s in this study to predict decompensated cirrhosis, the PPV was 97.9% while the NPV was 40.4%, which was not reliable for excluding decompensated cirrhosis. The AAR, APRI, and FIB-4 index also had similar predictive values as the ARFI (using cut-off values of 1.25, 0.84, and 5.00, the PPV were 89.8%, 83.5%, and 87.7%, respectively). This study explored the combined diagnosis of decompensated cirrhosis with ARFI and serum markers. The results showed that the combined assessment using ARFI+AAR, ARFI+APRI, and ARFI+FIB-4 did not further improve the accuracy of decompensated cirrhosis diagnosis compared to the diagnostic accuracy of ARFI alone.

There were several limitations in the current study. First, the number of enrolled patients was small. Second, the scores of patients in CP classes B and C were close, which may have affected the results. In future studies, the sample size should be increased and multi-center experiments should be performed.

In conclusion, we identified relationships between the ARFI, AAR, APRI, and FIB-4 index and the CP grade. The accuracy of ARFI in predicting decompensated cirrhosis was higher than that of the AAR, APRI, and FIB-4 index. Compared to the ARFI, the combined assessment using the ARFI+AAR, ARFI+APRI, and ARFI+FIB-4 did not further improve the accuracy of diagnosing decompensated cirrhosis. This study identified a convenient non-invasive diagnostic method for predicting decompensated cirrhosis in CHB patients in clinical practice.

## AUTHOR CONTRIBUTIONS

Wei L and Chen L conceived and designed the study. Wei L and Bao Z conducted the study. Lin X provided clinical information. Wei L and Xu X analyzed the data. Wei L wrote the manuscript. Ye Z was responsible for the revision of the manuscript. All authors read and approved the final version of the manuscript.

## Figures and Tables

**Table 1:** Gender and age of patients in each Child-Pugh class group.

	Child-Pugh A (n=24)	Child-Pugh B (n=45)	Child-Pugh C (n=35)
Gender (male/female)	19/5	37/8	28/7
Average age (years)	49.71±12.27	53.02±12.63	54.43±10.66
Body mass index(kg/m^2^)	22.91±2.24	22.44±2.31	22.35±2.57

**Table 2:** Analysis of the correlation between the ARFI, AAR, APRI, and FIB-4 index and the Child-Pugh class.

Indicators	Child-Pugh			F/χ^2^	*p*	Correlation	
	Child-Pugh A (n=24)	Child-Pugh B (n=45)	Child-Pugh C (n=35)			r_s_	*p*
ARFI	2.27±0.51	3.00±0.59*	3.13±0.55*	18.93	0.000	0.457	0.000
AAR	1.00 (0.71)	1.45 (0.93)*	1.61 (1.14)*	10.51	0.005	0.301	0.002
APRI	1.46 (2.36)	1.96 (2.67)*	2.44 (2.03)*	6.37	0.041	0.23	0.019
FIB-4	3.63 (4.38)	5.99 (6.53)*	8.73 (6.58)*	14.09	0.001	0.354	0.000

Comparison with Child-Pugh A *p<0.05.

**Table 3:** Diagnostic performance of the ARFI, AAR, APRI, and FIB-4 index in predicting decompensated cirrhosis.

Indicator	AUC	95%CI	Cut-off	Sensitivity (%)	Specificity (%)	PPV (%)	NPV (%)
ARFI	0.841	0.756-0.905	2.99	57.5	95.8	97.9	40.4
AAR	0.707	0.610-0.792	1.25	66.3	75.0	89.8	40.0
APRI	0.664	0.565-0.753	0.84	88.8	41.7	83.5	52.6
FIB-4	0.736	0.640-0.818	5.00	71.3	66.7	87.7	41.0

PPV: positive predictive value, NPV: negative predictive value.

**Table 4:** The value of ARFI combined with AAR, APRI, or FIB-4 index in diagnosing decompensated cirrhosis.

	AUC	95%CI	Sensitivity (%)	Specificity (%)	PPV (%)	NPV (%)
ARFI+AAR	0.858	0.776-0.919	73.8	83.3	93.7	48.8
ARFI+APRI	0.847	0.763-0.910	58.8	95.8	97.9	41.1
ARFI+FIB-4	0.874	0.795-0.931	73.8	87.5	95.2	50.0

PPV: positive predictive value, NPV: negative predictive value.

**Table 5:** Comparison of ARFI+AAR, ARFI+APRI, ARFI+FIB-4, and ARFI for the diagnosis of decompensated cirrhosis.

	AUC	difference	95%CI	Z	*p*
ARFI	0.841	0.017	-0.037-0.070	0.608	0.543
ARFI+AAR	0.858				
ARFI	0.841	0.006	-0.007-0.018	0.851	0.395
ARFI+APRI	0.847				
ARFI	0.841	0.033	-0.011-0.078	1.469	0.142
ARFI+FIB-4	0.874				
